# The characterization of an intestine-like genomic signature maintained during Barrett’s-associated adenocarcinogenesis reveals an NR5A2-mediated promotion of cancer cell survival

**DOI:** 10.1038/srep32638

**Published:** 2016-09-02

**Authors:** Shane P. Duggan, Fiona M. Behan, Murat Kirca, Abdul Zaheer, Sarah A. McGarrigle, John V. Reynolds, Gisela M. F. Vaz, Mathias O. Senge, Dermot Kelleher

**Affiliations:** 1 Department of Medicine, Division of Gastroenterology, University of British Columbia, 2775 Laurel Street, Vancouver, British Columbia, Canada; 2 Life Science Institute, 2350 Health Sciences Mall, Vancouver, British Columbia, Canada; 3 Department of Clinical Medicine, Institute of Molecular Medicine, Trinity College Dublin, St James’ Hospital, Dublin, Ireland; 4 Department of Gastroenterology, St James’ Hospital, Dublin, Ireland; 5 Department of Surgery, Institute of Molecular Medicine, Trinity College Dublin, St James’ Hospital, Dublin 8, Ireland; 6 Medicinal Chemistry, Trinity Translational Medicine Institute, Trinity College Dublin, the University of Dublin, St James’ Hospital, Dublin 8, Ireland

## Abstract

Barrett’s oesophagus (BO), an intestinal-type metaplasia (IM), typically arising in conjunction with gastro-oesophageal reflux disease, is a prominent risk factor for the development of oesophageal adenocarcinoma (OAC). The molecular similarities between IM and normal intestinal tissues are ill-defined. Consequently, the contribution of intestine-enriched factors expressed within BO to oncogenesis is unclear. Herein, using transcriptomics we define the intestine-enriched genes expressed in meta-profiles of BO and OAC. Interestingly, 77% of the genes differentially expressed in a meta-profile of BO were similarly expressed in intestinal tissues. Furthermore, 85% of this intestine-like signature was maintained upon transition to OAC. Gene networking analysis of transcription factors within this signature revealed a network centred upon NR5A2, GATA6 and FOXA2, whose over-expression was determined in a cohort of BO and OAC patients. Simulated acid reflux was observed to induce the expression of both NR5A2 and GATA6. Using siRNA-mediated silencing and an NR5A2 antagonist we demonstrate that NR5A2-mediated cancer cell survival is facilitated through augmentation of GATA6 and anti-apoptotic factor BCL-XL levels. Abrogation of NR5A2-GATA6 expression in conjunction with BCL-XL co-silencing resulted in synergistically increased sensitivity to chemotherapeutics and photo-dynamic therapeutics. These findings characterize the intestine-like signature associated with IM which may have important consequences to adenocarcinogenesis.

Barrett’s oesophagus (BO) is present in 10–20% of patients with gastro-oesophageal reflux disease (GORD), which itself has prevalence estimates between 18.1–27.8% of the population^1^,^2^. Both BO and GORD are important risk factors in the development of oesophageal adenocarcinoma (OAC)^3^,^4^,^5^,^6^,^7^,^8^,^9^. While GORD is a risk factor for BO development, there is limited evidence that anti-reflux measures, including PPIs and surgery, abrogate the risk of cancer development from BO. Hence it is likely that components of the intestinal nature of Barrett’s metaplasia may be crucial for oesophageal adenocarcinogenesis. This study aims to fully define the genomic intestinal signature of BO and to identify factors that persist in the process of oesophageal adenocarcinogenesis. It has been suggested that BO arises through the differentiation of either oesophageal stem cells or gastric cardia-progenitor cells present within the lower oesophageal squamous epithelium in response to chronic GORD^3^,^10^,^11^,^12^. Some similarities between intestinal metaplasia (IM) and the developmental processes of normal intestinal tissue have emerged centring upon intestinal-type transcription factors (TFs) such as CDX2^13^,^14^,^15^, and recently FOXA2^16^,^17^,^18^. These factors have been observed to regulate the expression of histological intestinal markers such as sucrose isomaltase, villin and mucins, but their contributions to adenocarcinogenesis are unclear^14^,^15^,^19^,^20^,^21^. Thus, the extent of the similarities between the processes of differentiation utilized in IM and normal intestinal tissues remains to be examined. Additionally, the mechanistic links between GORD, the development of IM and GORD-mediated promotion of adenocarcinogenesis have also not been fully elucidated.

Genetic instability is a hallmark of lower oesophageal metaplasia including intestinal, junctional and fundic^22^. However, of the three metaplastic types, the intestinal type metaplasia of BO is most prominently associated with progression to dysplasia and adenocarcinoma^4^,^6^,^22^,^23^,^24^,^25^. Thus the intestinal nature of BO, in conjunction with GORD, genetic influences, epigenetic factors and genomic instability may potentially contribute to the development of OAC. BO and normal intestinal tissue display apparent ultra-structural similarities upon histological examination including the presence of mucin-secreting goblet cells, villi, surface microvilli and a similar immune complement^26^,^27^,^28^,^29^,^30^. Whether this similarity holds true at a molecular level is also unknown.

Herein, by performing functional genomic analysis of oesophageal, duodenal and colonic tissues we have defined a signature of genes characteristic of intestinal tissue identity, by reference to normal oesophageal tissue. We further analysed the expression of this signature in clinical tissues and genomic meta-profiles of BO, OAC, and other cancer types to delineate the nature of the genes expressed with particular emphasis on transcription factors (TFs), as ultimate regulators of tissue identity and developmental processes. We demonstrate that a specific pattern of TFs associated with intestinal tissue are also observed in BO and the resultant OAC. This included TFs such as NR5A2, GATA6, FOXA2, SMAD6, NR1H4, NR1I2 and ARNTL2 and gene networking analysis suggested a central role for NR5A2 in this signature. Functional studies revealed a significant contribution of NR5A2 in mediating transcriptional responses to simulated GORD events and promoting cellular survival of OAC cells through modulation of GATA6 and BCL-XL levels. Jointly, these findings implicate NR5A2 in the process of oesophageal metaplasia and suggest its potential as a therapeutic target in OAC, in addition to standard chemotherapeutic modalities.

## Results

### Defining an intestine-enriched signature by reference to normal oesophageal tissue

**Figure 1:**
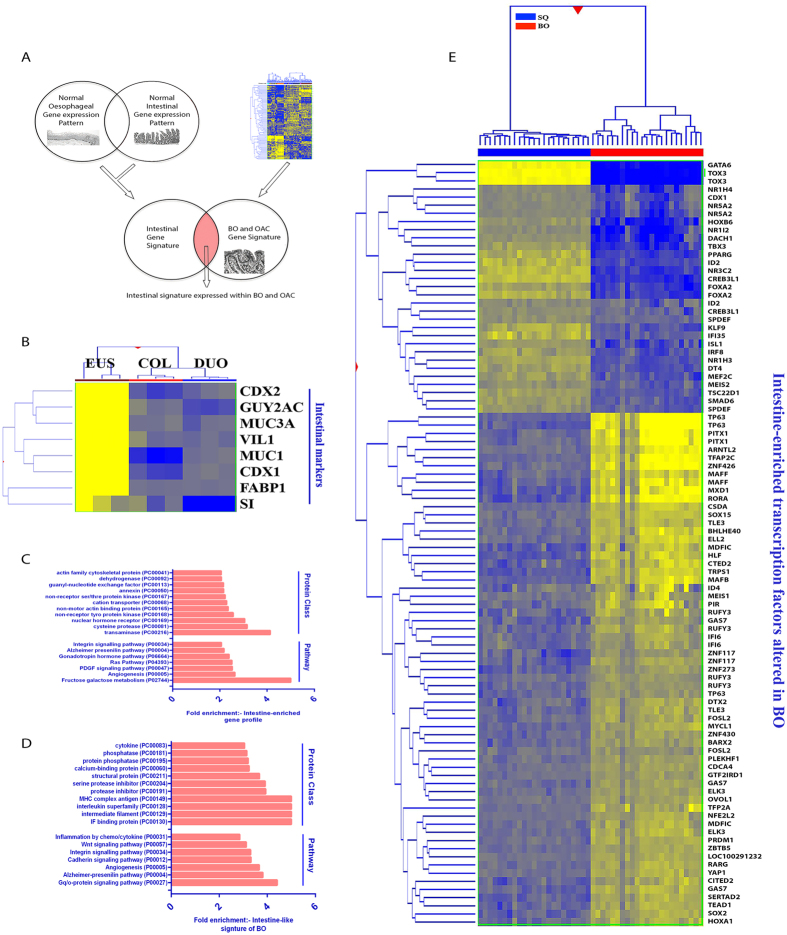
Defining the intestine-like signature of BO and its constituent transcription factors. (**A**) Inter-comparisons of normal intestinal and oesophageal tissue expression patterns by gene expression microarray compared with the gene signature of BO and associated adenocarcinoma (OAC). (**B**) Heat map showing differential expression of known intestinal type markers in duodenal, colonic and oesophageal tissues as defined by this comparison with a significance cut-off of p < 0.001 in Mann-Whitney test. The over-represented protein class and pathway enrichment of the intestinal gene profile (**C**) and the intestinal signature of BO (**D**) using pantherdb classifications and enrichments. (**E**) Transcriptions factors of the intestinal signature are altered in BO. Hierarchical heat-map clustering of 74 transcription factors, defined as intestinal components of BO viewed through GEM study of Ostrowski *et al.* (Mann-Whitney p < 0.001, FC > ± 1.5). Heat maps:- Yellow (low expression, FC > −1.5), Blue (high expression, FC > + 1.5). Legend:- SQ- normal squamous oesophageal tissue; BO- Barrett’s oesophagus tissue.

Gene expression microarray (GEM) analysis of histologically normal oesophageal (n = 3), colonic (n = 3) and duodenal (n = 3) tissues was performed using HG133+2 Affymetrix chips measuring 47,000 individual transcripts as outlined in Materials and Methods. The resulting GEM data was used to define an intestinal gene signature representative of these tissues through statistical inter-comparisons (Fig. 1A). This signature was composed of genes similarly altered in duodenal and colonic tissue by comparison to normal oesophageal tissue. It was composed of 2861 unique up- and down-regulated genes (FC1.5, p < 0.001, Mann-Whitney (MW) test), which included conventional intestinal markers VIL1, GC-C, MUC2, MUC3 and CDX1/2, supporting the approach (Fig. 1B). Some of the major gene classes over-represented in this intestinal signature included genes coding for transaminases (e.g., GPT, GPT2, OAT, TAT), cysteine proteases (e.g., CASP6-7, BMP8B, CTSO, cathespin -S, -11, -L2 and PDCD6) nuclear hormone receptors (e.g., NR3C2, RARB, HNF4A, NR1H3) and non-receptor tyrosine protein kinases (CDK7, 10 and 18; ADAM15, MAP3K13, LIMK2) (Fig. 1C). The leading statistically over-represented biological process and molecular functions included digestive tract mesoderm development (e.g., HOXA2-5, HOXB5-7 and PDX1) and exopeptidase activities (e.g., AOPEP, ACE, ERAP, GGH, seperase), all reflective of a distinct intestinal signature and gastrointestinal function (Fig. 1C). However, the largest overall gene class was defined as nucleic acid binding proteins, with 266 unique genes/proteins that demonstrate sequence specific DNA-binding transcription factor activity (GO:0003700).

### An intestine-like signature within BO contains a selective node of cooperative transcription factors centred upon NR5A2

We next compared the intestinal profile defined above with a shared meta-expression profile of BO tissue to determine the underlying molecular pattern that results in the phenotypic similarities between this intestinal metaplasia and that of normal intestinal tissue architecture. This meta-profile of BO (989 genes) was generated through statistical re-analysis of the GEM studies of Kimchi *et al.* (GSE1420)^31^, Ostrowski *et al.* (GSE36223)^32^ and Wang *et al.* (GSE26886)^33^, using HGU133A annotation data mask to provide a common platform of gene/probe numbers. This approach allowed a common statistical approach between all platforms (Mann-Whitney test at p < 0.001, Supplementary Fig. S1). An inter-comparison showed that 77.7% (or 768 genes) of all differentially expressed transcripts in the meta-profile of BO are similarly altered in intestinal tissues from the intestinal gene profile (Mann-Whitney test at p < 0.001, S1 Table). The remaining differentially expressed genes within the BO meta-profile, that were not observed to be altered between oesophageal and intestinal tissues (22.3% or 220 genes), were defined as non-intestinal components of the BO signature. The intestine-like signature of BO was comprised of 26.8% (769 genes) of all possible intestine-enriched genes (2861 genes) as defined by the above comparison between oesophageal and intestinal tissues, emphasizing that a specific subset of the intestinal molecular make-up was expressed within BO.

The over-represented gene classes that were enriched as intestinal components of BO consisted of intermediate filaments and binding proteins (e.g., KRT5, 8, 15 and 37, Periplankin, PKP1, EVPL, SOS1), interleukin superfamily members (e.g., RGS12, IL12A, IL36G, IL20, IL1RN), major histocompatibility complex antigens (e.g., HLA-F, HLA-DRB5, HLA-DQB2, ODC1) and protease inhibitors (e.g., CASP6-7, PRKD1, KLK7, CST6, SERPINB2) (Fig. 1D). Over-represented pathways within the intestine-like signature of BO included Gq/Go alpha-protein signalling (e.g., RGS12, PRKCH and RHOC), angiogenesis (e.g., PDGFA, NOTCH2, PRKCA and MCAM) and Wnt signalling pathways (e.g., NOTCH2, FZD3, FRZB, NR5A2, CDH17). Comparatively, gene classes from functional groups such as exopeptidases and transaminases, as previously associated with intestinal tissue function from the intestinal gene profile, were not enriched within the BO meta-signature.

**Figure 2:**
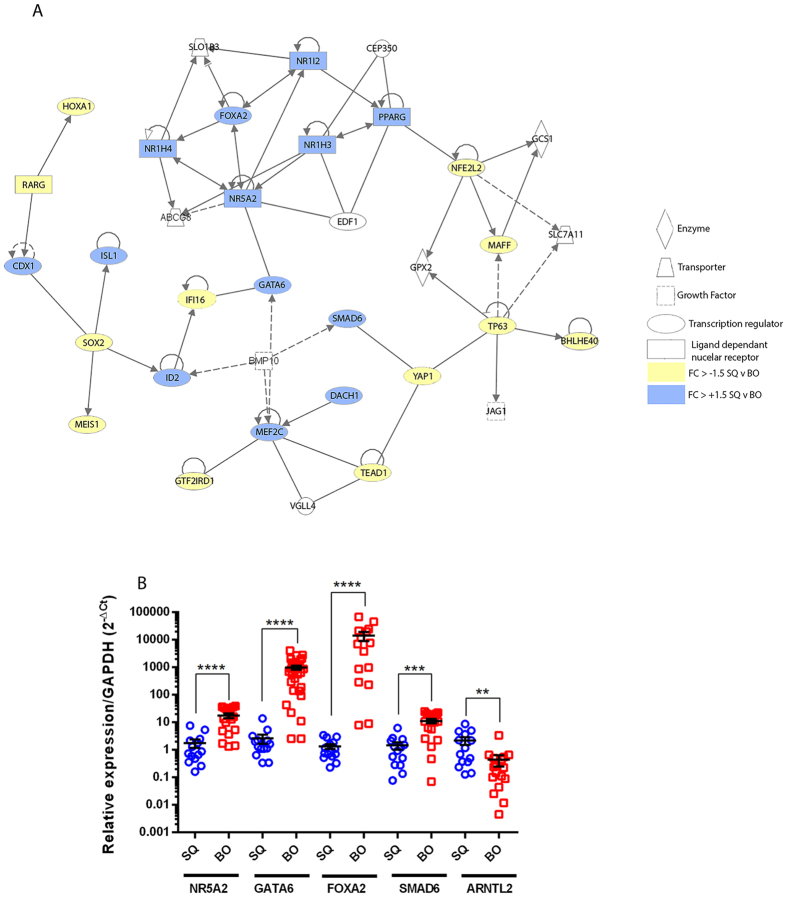
Gene networking and validation of intestine-enriched TF expression in BO. (**A**) Gene networking of the 74 intestinal TFs expressed in BO from Fig. 1C using Ingenuity Pathway Analysis (IPA) identified a single core network of 26 TFs with NR5A2 central to the majority of the interactions. (**B**) Clinical expression of NR5A2 (FC = 17.85), FOXA2 (FC = 141.62), SMAD6 (FC = 11.27), GATA6 (FC = 262.06) and ARNTL2 (FC = 2.180126) mRNA in BO patient (n = 17 samples with 3 technical replicates) and control cohorts (n = 17 with 3 technical replicates) by real time RT-PCR, all changes were significant to p < 0.00001****p < 0.0001*** and p < 0.001** in two-tailed non-parametric Mann-Whitney testing (error bars display SEM). Legend:- yellow fill- down regulated in BO; Blue fill- up regulated in BO; No fill- not present in signature. SQ- normal squamous oesophageal tissue (blue); BO- Barrett’s oesophagus tissue (red).

Nucleic acid binding factors represented a significant proportion (16%) of the overall genes altered within this intestine-like signature of BO (n = 123/769 genes, GO Mol.Fn), with 74 sequence specific regulatory TFs identified (Fig. 1E), including TFs such as CDX1, HOXB7, HOXB6, TP63, NR5A2, GATA6, FOXA2 and TOX3. Gene networking of these TFs defined a potential core network of 26 TFs, centred upon NR5A2, GATA6 and FOXA2 (Fig. 2A), with the remaining TFs distributed among less significantly populated networks. Of note, substantially greater levels of NR5A2 (FC = 7.85; MW p < 0.0001), FOXA2 (FC = 141.62; MW p < 0.0001), GATA6 (FC = 262.06; MW p < 0.0001) and SMAD6 (FC = 11.27; MW p < 0.001) transcripts were validated in BO biopsies (n = 17 samples and 3 technical replicates) relative to normal oesophagus (n = 17 samples and 3 technical replicates) in follow-up real time RT-PCR validation studies with significance determined by non-parametric (Mann-whitney) testing appropriate for gene expression data (Fig. 2B).

### The BO-associated intestine-like signature is maintained in a meta-profile of OAC

**Figure 3:**
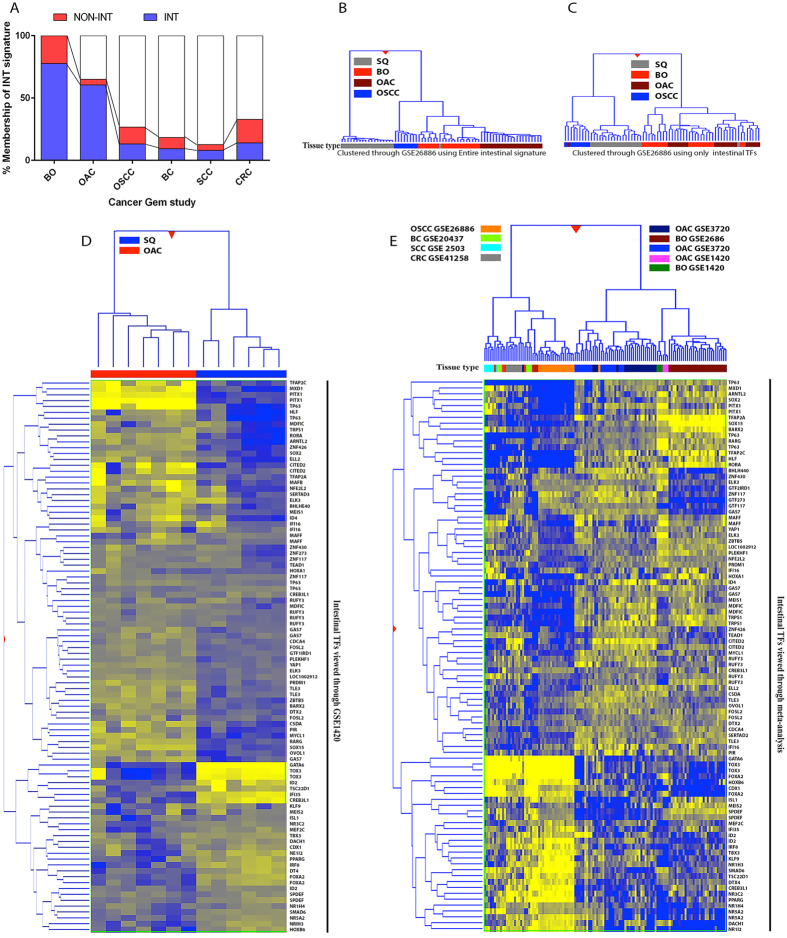
The intestine-like genomic signature of BO is specific to, and maintained during, oesophageal adenocarcinogenesis. (**A**) Proportion of the intestine-like signature of BO expressed in GEM studies of OAC, OSCC, BC, SCC and CRC expressed as percentage of total altered genes. Dendrograms resulting from hierarchical clustering of the entire intestine-like signature of BO (**B**) and clustering of only the 74 constituent transcription factors (**C**) using GSE26886. Less clear separation of tissue types is noted when clustering using intestinal TF expression. (**D**) Hierarchical clustering of intestinal TFs expressed in OAC by comparison to squamous tissue using GSE1420 study. (**E**) Hierarchical clustering of multiple cancer lesions, normalized to respective control tissues, (from A) demonstrating distinct expression of the intestine-like TF signature in BO and OAC GEM studies. All studies performed using the same analysis platform and examined by Mann-Whitney testing at p < 0.001 and fold change greater than ± 1.5. Heat maps:- Blue-upregulated; yellow-down-regulated; dark shades-midpoint; Legend:- INT:- Present in the Intestine-like signature of BO; NON-INT:- Not present in the intestine-like signature of BO; SQ- normal squamous oesophageal tissue; BO- Barrett’s oesophagus tissue; OAC-oesophageal adenocarcinoma; OSCC- oesophageal squamous cell carcinoma; BC- Breast Cancer; SCC- skin squamous cell carcinoma; CRC- colorectal cancer.

A combined meta-profile of OAC (1078 genes, Mann-Whitney, p < 0.001) developed utilizing GEM studies GSE1420^31^, GSE3720^34^ and GSE26886^33^, was next utilized to examine the expression of the BO-associated intestine-like signature in OAC. Interestingly, 85% (652 genes) of the intestinal signature of BO (769 genes) was similarly expressed in the meta-profile of OAC (60.5% of total altered genes in OAC, 652/1078 genes). Comparatively, the non-intestinal signature of BO was greatly reduced, with only 45 genes or 20.3% of the non-intestinal meta-signature of BO (45/220 genes) still altered in OAC tissue (Fig. 3A and S1 Table, associative χ2-p < 0.0001). The non-intestinal factors maintained upon OAC formation included 8 TFs (e.g., MAF, ESRP1, HOXA10, ZNF512B), 13 signalling factors (e.g., GDF15, APP, TFF1, TSPAN6) and 8 enzyme modulators (e.g., TIMP1, RGS5, CSTA, VAV3). Conversely, the cohort of non-intestinal genes that were lost upon transition to OAC contained 7 TFs (e.g., ZNF395, ETV5, NR4A3, JARID2) and 13 regulators of cell proliferation (e.g., NR4A3, FGFR1 and 4; ITGB1 and 4; DUSP22, ST7) with the remainder unclassified due to lack of functional enrichment.

We next examined the specificity of the intestine-like signature of BO in other cancer types. Underlining the differing aetiology of oesophageal squamous cell carcinoma (OSCC) was the lack of the intestinal signature in GEM analysis (GSE26886) of this malignancy (13%, χ2-p < 0.00001, Fig. 3A). In GEM data (GSE41258) of colorectal cancer (CRC) a lower proportion of the BO associated intestinal signature (14%, χ2p < 0.00001) was expressed by comparison to normal colonic tissue. Notably, loss of the intestinal factor CDX2 in CRC has been associated with progression, increased tumour grade and high mortality^35^. Additionally, NR5A2 expression is derived from within a DNA methylation valley observed to be hyper-methylated in CRC resulting in reduced expression in some studies^36^. Similarly, expression of the intestinal signature was considerably lower in GEM studies of skin squamous cell carcinoma (8.8%, χ^2^-p < 0.00001, GSE2503) and breast cancer (9.5%, χ^2^-p < 0.00001, GSE20437) (Fig. 3A).

### Expression of Intestine-like TF signature in OAC

Hierarchical gene and sample clustering of BO and OAC using the intestine-like TF signature of BO in GSE26886, could not accurately separate these tissue types (Fig. 3B, C), indicative of the similarity in the expression patterns of these TFs between BO and OAC. Importantly, of the 74 TFs defined from the intestinal signature of BO, 47 were still altered in OAC tissue (Fig. 3D), both up-regulated (e.g., KLF9, HOXB6, HOXB7, TOX3 and SOX4) and down-regulated (e.g., PITX1 MAF, MXD1 and BARX1). Notably, core networked TFs of the intestine-like signature of BO such as NR5A2, GATA6 and FOXA2 were still strongly up-regulated in OAC tissue. Furthermore, the expression of this intestine-like TF signature could clearly be observed in multiple GEM studies of BO and OAC, and largely absent from GEM studies of squamous cell carcinoma, CRC and breast cancer (Fig. 3E).

**Figure 4:**
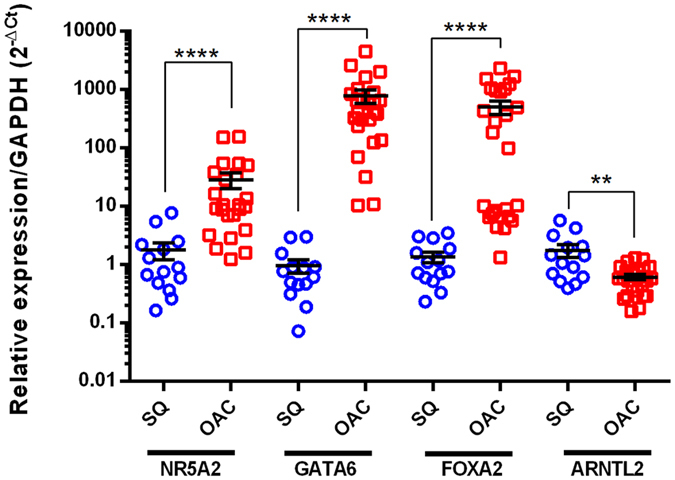
Intestine-enriched TFs are over-expressed in OAC tissue. Clinical expression of NR5A2 (FC = 12.4534), GATA6 (FC = 375.97) FOXA2 (FC = 198.34), and ARNTL2 (FC = 2.180126) mRNA in OAC patient (n = 23 samples with 3 technical replicates) and control (n = 17 with 3 technical replicates) cohorts by real time RT-PCR, all changes were significant to p < 0.00001****p < 0.0001***p < 0.001** in two-tailed non-parametric Mann-Whitney testing (error bars display SEM). SQ- normal squamous oesophageal tissue (blue); OAC Oesophageal adenocarcinoma tissue (red).

Independent real time RT-PCR validation experiments demonstrated the increased expression of NR5A2 (FC = 12.4534; MW p < 0.0001), GATA6 (FC = 375.97; MW p < 0.0001) and FOXA2 (FC = 198.34; MW p < 0.0001) in a cohort of OAC patient tissues (n = 23 samples with 3 technical replicates) with significance determined by non-parametric testing appropriate for gene expression data (Fig. 4). Thus, the core TFs of this intestine-like signature associated with BO is maintained throughout adeno-carcinogenesis and may provide some of the molecular components for this transformation and its survival in the lower oesophagus.

### Simulated reflux events cumulatively induce intestine-enriched TFs NR5A2 and GATA6

**Figure 5:**
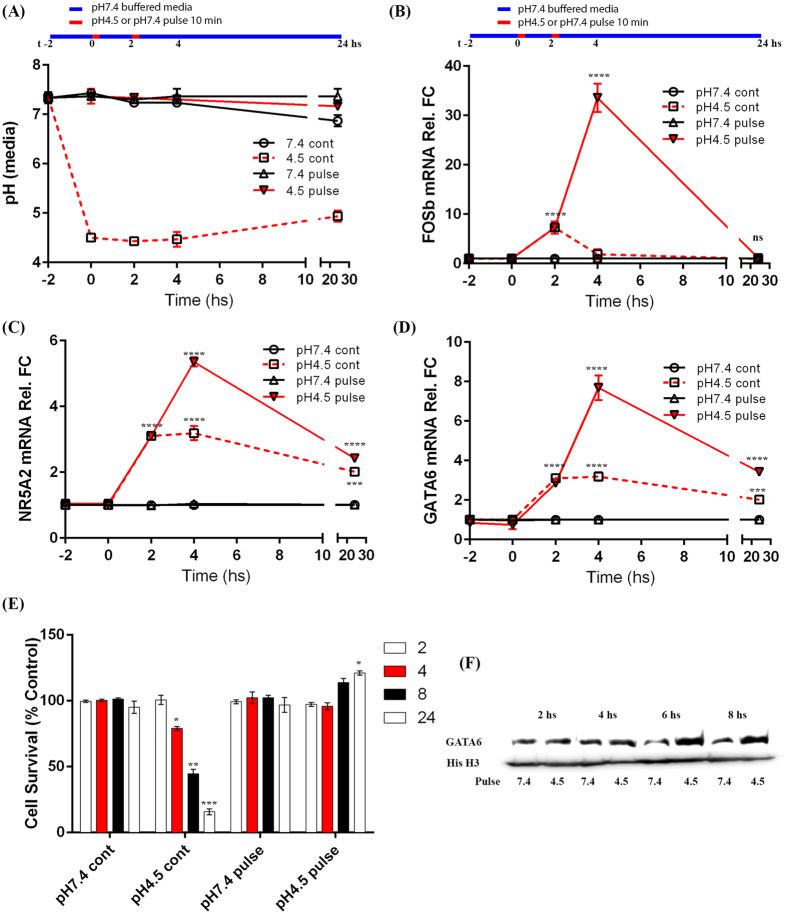
Induction of NR5A2 and GATA6 expression by pulsed gastric acid in oesophageal cells. SKGT4 cells were exposed to simulated reflux events, 10 min in duration, using acidified cell culture media at pH4.5 or non-acidified media at pH7.4 (control) followed by incubation at pH7.4 for 2 hs prior to repeating the reflux event. (**A**) pH levels monitored throughout experiment and 2 hs prior to initial insult demonstrates only minor fluctuations in intended pH. (**B**) FOSb (positive control), (**C**) NR5A2 and (**D**) GATA6 mRNA levels in SKGT4 cells exposed to differing acidic environments (continuous (cont) or pulsatile (pulse) (10 min) at pH4.5 or pH7.4) examined by real time RT-PCR at t-2, t0, t+2, t+4 and t+24 hs. (**E**) Cell viability following acidic treatments in SKGT4 cells measured by MTT assay following 2 repeated exposures. (**F**) Induction of GATA6 protein expression following exposure to pulsatile low pH4.5 following 2 repeated exposures measured over 8 hs using histone H3 blotting as loading control (image details live zoomed cropped exposure of GATA6 and loading control bands as capture by CCD imager). Significance was achieved by two-tailed non-parametric Mann-Whitney testing for RT-PCR data and students t-test in viability and Western blotting experiments, p < 0.00001****, p < 0.0001***, p < 0.001**, p < 0.01*, (n = 3 in all experiments and error bars represent SD).

Episodic exposure to gastric acid, as associated with GORD, has consistently been linked with the development of both BO and OAC^5^,^37^. However, the mechanistic molecular links between GORD, the development of IM and subsequent support for oncogenesis remain elusive. Therefore we next examined the ability of simulated reflux to alter the expression of NR5A2, GATA6 and FOXA2, the core BO- and OAC-associated intestine-enriched TFs identified by gene networking above. Pulsatile reflux was simulated using 10 min exposure of SKGT4 OAC cells in culture to unbuffered acidified culture media (pH4.5) media followed by return to culture media at pH7.4 during rest period (Fig. 5A). Control cells were also pulsed with non-acidified media at pH7.4 for direct comparison. Samples for mRNA analysis and pH monitoring were taken prior to the start of the experiment and at various time points throughout (t = −2, 0, 2, 4 and 24 hs). The pH of the media did not vary greater than 0.2 pH from intended pH except in the case of continuous exposure of cells in culture to pH4.5, which had increased to pH5 at 24 hs (Fig. 5A). This was most likely due to the length of the incubation and the observed cell death that occurs in continuous exposure to such pHs.

Pulsed exposure of SKGT4 cells to gastric acid-treated culture media resulted in FosB (positive control), NR5A2 and GATA6 mRNA and protein induction, with the greatest levels achieved by repeated pulsed exposure (10 min every 2 hs) at pH4.5 (FC = 33.56, FOSb; FC = 5.31, NR5A2; FC = 7.8, GATA6; at 4 hs MW p < 0.001) simulating the *in-vivo* reflux episodes (Fig. 5B–D). No overt effects on FOXA2 expression (FC = 0.92, MW p = 0.34) or cell viability was observed during pulsatile exposures (Fig. 5E). NR5A2 and GATA6 mRNA levels remained significantly higher in OAC cells 24 hs post-pulsed exposure to pH4.5 (NR5A2 = 3.85FC; GATA6 = 3.5FC; 24 hs, MW p < 0.001, Fig. 5C,D) by comparison to FOSb levels, which returned to basal levels (FC = 1.02; FOSb) 24 hs following acid pulsing (Fig. 5B). Induction of GATA6 protein was confirmed in OAC cells by Western blotting using His H3 as blotting control (Fig. 5F). Thus, repeated chronic exposure to low pH can cumulatively induce prominent transcription factors of the intestine-like signature of BO and OAC.

### NR5A2 regulates OAC cell survival through modulation of GATA6 expression levels

**Figure 6:**
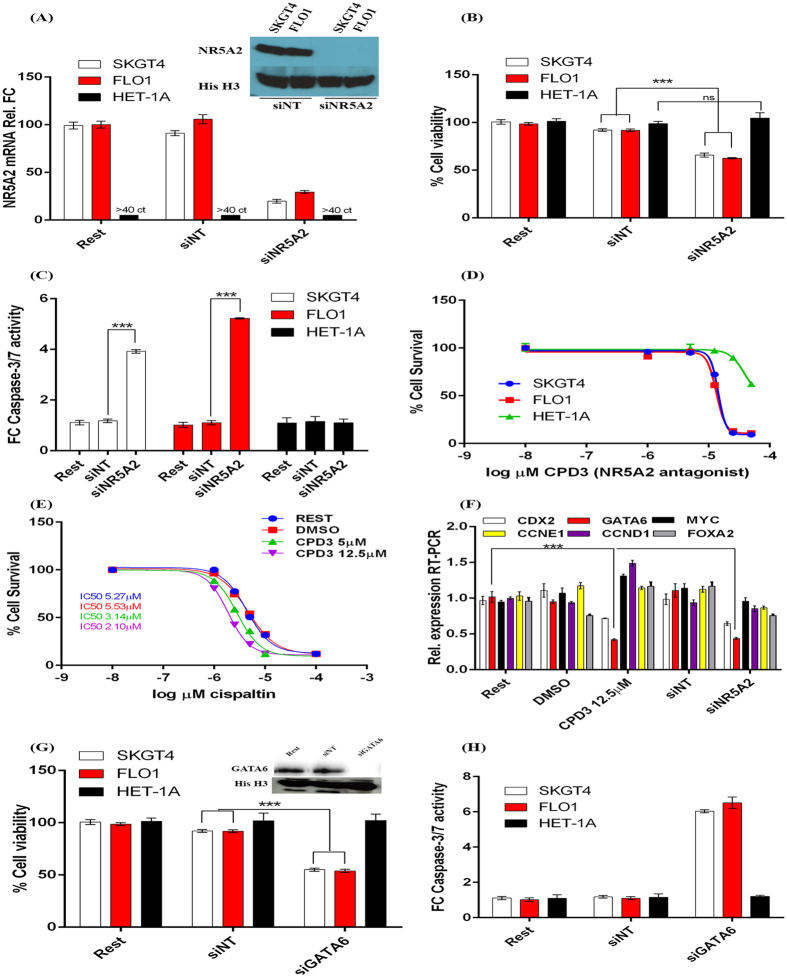
Silencing and antagonism of NR5A2 affects OAC cell survival and response to cisplatin. (**A**) NR5A2 levels following silencing by siRNA in SKGT4 and FLO1 OAC cells and HET-1A squamous cells by real time RT-PCR and Western blotting (inset of cropped image from Fig. S3). No detectable levels of NR5A2 in HET-1A cells and thus is represented by ct cycles >40. (**B**) Cell viability by MTT assay following siRNA-mediated silencing of NR5A2. (**C**) caspase-3/7 activity following siRNA-mediated inhibition of NR5A2 as measured by DEVD cleavage assay in OAC cells. (**D**) Cell survival following treatment of oesophageal cells with the NR5A2 antagonist CPD-3 as measured by MTT assay in oesophageal cell lines. (**E**) Cell survival following exposure of SKGT4 OAC cells to a concentration curve of cisplatin in conjunction to treatment with DMSO 0.1% (vehicle), CPD-3 at 5 μM and 12.5 μM. (**F**) Real time RT-PCR expression levels of cell cycle and closely networked genes following either NR5A2 silencing or treatment with CPD-3 at 12.5 μM in SKGT4 cells. (**G**) Cell viability by MTT assay following siRNA-mediated silencing of GATA6. Inset:- Western blotting of GATA6 expression following siRNA-mediated silencing in SKGT4 cells (image details live zoomed cropped exposure of GATA6 and loading control bands as capture by CCD imager). (**H**) caspase-3/7 activity following siRNA-mediated inhibition of GATA6 in OAC cells. Significance was achieved by non-parametric Mann-Whitney testing for RT-PCR data and students t-test in viability and Western blotting experiments, p < 0.00001****, p < 0.0001***, p < 0.001**, p < 0.01*, (n = 3 in all experiments and error bars represent SD). Legend:- Rest-resting cells; siNT- non-targeting siRNA; CPD3- NR5A2 antagonist; DMSO-vehicle; FC-fold change.

NR5A2 was the dominant interactant in gene networking analysis performed above and thus was of primary interest. Silencing of NR5A2 by siRNA-mediated inhibition in SKGT4 and FLO1 cells resulted in substantial loss of NR5A2 expression (Fig. 6A), significant growth arrest (66% and 62% respectively, n = 3, t test p < 0.001, Fig. 6B) and increased caspase-3/7 activity (FC = 3.9 and FC = 4.2, n = 3, t test p < 0.005, Fig. 6C). NR5A2 was not expressed in HET-1A cells which are representative of normal oesophageal epithelial squamous cells. Furthermore, transfection of HET-1A cells with siNR5A2 did not alter growth or caspase cleavage in HET-1A cells. These findings were replicated following treatment of OAC cell lines with the NR5A2 antagonist CPD-3, resulting in substantial growth arrest (Fig. 6D) and increased sensitivity to cisplatin in OAC cell lines (Fig. 6E). Loss or antagonism of NR5A2 expression did not affect levels of CCND1, CCNE, FOXA2 or MYC (Fig. 6F), but did result in significantly reduced levels of GATA6 expression (n = 3, MW p < 0.001), a closely networked gene that was also responsive to acidic exposure. Interestingly, levels of CDX2 were also reduced (n = 3, MW p < 0.01) under either antagonism or inhibition of NR5A2, but not to the same extent as observed for GATA6 (Fig. 6F). Inhibition of GATA6 by siRNA-mediated silencing similarly resulted in reduced cell survival in SKGT4 (55%, n = 3, t test p < 0.001) and FLO1 (45%, n = 3, t test p < 0.001) cells (Fig. 6G) and increased caspase-3/7 activity (Fig. 6H). These effects were not observed in GATA6 negative HET-1A squamous oesophageal cells (n = 3, t test p = ns0.9493).

**Figure 7:**
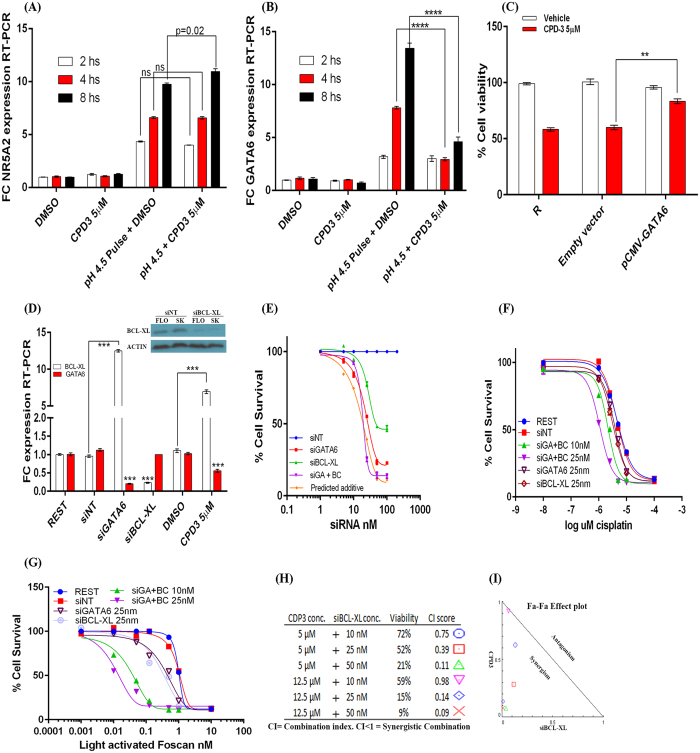
NR5A2 controls OAC cell survival through modulation of GATA6 and BCL-XL levels. NR5A2 (**A**) and GATA6 (**B**) mRNA levels in SKGT4 cells pre-treated with the NR5A2 antagonist (CPD-3) following acid pulsing. (**C**) Cell viability, measured by MTT, of SKGT4 cells transfected with either empty vector or rescued by GATA6 expression vector (pCMV-GATA6) following treatment with CPD-3. (**D**) Expression levels of GATA6 and BCL-XL following silencing of GATA6 and BCL-XL or treatment with CPD-3; inset- Western blotting of BCL-XL and actin (loading control) expression following transfection with siNT or siBCL-XL in FLO1 and SKGT4 cells (cropped image from Fig. S3). (**E**) SKGT4 OAC cell survival following silencing of GATA6 (siGATA6), BCL-XL (siBCL-XL) or co-silencing of both GATA6 and BCL-XL (siGA+BC) over a range of siRNA concentrations (predicted additive affect is shown in orange). Dose response curves of cisplatin (**F**) and light activated PDT agent Foscan (**G**) in SKGT4 OAC cells co-silenced for GATA6 and BCL-XL at 10 nm and 25 nm siRNA as measured by MTT assay. Data is normalised to respective untreated control cells, cells transfected siNT at 10 nm or 25 nm, siGATA6 alone or siBCL/XL alone. (**H**) Combination Index (CI) score of synergistic cell survival following combined abrogation of NR5A2 and BCL-XL function, by antagonist and siRNA-mediated silencing respectively, in SKGT4 cells as measured by MTT assay. (**I**) Isobologram of (H). Significance was achieved by two-tailed non-parametric Mann-Whitney testing for RT-PCR data and students t-test in viability and Western blotting experiments, p < 0.00001****, p < 0.0001***, p < 0.001**, p < 0.01*, (n = 3 in all experiments and error bars represent SD). Synergism was defined by Chou-Talalay combination index scoring in Compusyn software. Legend:- Rest- resting cells; CPD-3- NR5A2 antagonist; Pulse- pulsatile repeated exposure 10 mins; pCMV-XL6- GATA6 expression vector; siNT- non-targeting siRNA; DMSO- vehicle; siGA+siGA- equimolar concentrations of siBCL-XL and siGATA6 combined.

As both NR5A2 and GATA6 are so closely linked we further examined their potential inter-regulation. Importantly, pre-treatment of SKGT4 cells with 5 μM of the NR5A2 antagonist (CPD-3) 30 min prior to exposure to acidic pulse at pH4.5, resulted in reduced induction of GATA6 mRNA (Fig. 7A) at 4 and 8 hs (FC = 4.3, 8 hs, MW p < 0.0001), with no effects on pulse-mediated NR5A2 levels (Fig. 7B). This further supports an NR5A2-mediated regulation of GATA6 levels in OAC cells. GATA6 has been reported to regulate intestinal crypt proliferation^38^ and cancer cell survival^39^, and thus may be at least partially responsible for the observed growth arrest following CPD-3 treatment. To this end, partial recovery of CPD-3-mediated growth arrest was achieved (84%, p = 0.004) in SKGT4 cells transfected with a GATA6 overexpression vector by comparison to empty vector (Fig. 7C). These findings indicate that NR5A2 may affect OAC cell survival through regulating GATA6 expression levels, and suggests supportive routes to oncogenesis by expression of these factors during intestinal transformation in the oesophagus.

### Protective induction of anti-apoptotic factor BCL-XL in response to NR5A2 and GATA6 abrogation

Inhibition of either NR5A2 or GATA6 did not result in complete cell death in OAC cells, suggestive of a protective induction of anti-apoptotic mechanisms. During validation of GATA6 knockdown we demonstrated that inhibition of GATA6 resulted in significant up-regulation of the anti-apoptotic factor BCL-XL in SKGT4 cells (FC = 12.3, MW p < 0.001, Fig. 7D). Therefore we next examined whether greater cell death could be achieved by abrogation of the NR5A2-GATA6 axis in conjunction with the silencing of BCL-XL expression. Co-silencing of both BCL-XL and GATA6 was achieved by siRNA-mediated co-transfections in SKTG4 cells over a range of siRNA concentrations (1–100 nm each siRNA) resulting in potential synergistic effects (n = 3, CI = 0.67) on cell survival at 25 nM of each co-transfected siRNA as determined by Chou-Talalay combination index (CI) synergy score (Fig. 7E). This approach was utilized to further sensitize OAC cells to cisplatin (Fig. 7F) and the PDT agent Foscan (Fig. 7G). Similarly, silencing of BCL-XL in SKGT4 cells by siRNA-mediated silencing followed by treatment with CPD-3 (0.1–50 μM) at 72 hs also resulted in CI survival scores at 5 μM (n = 3, CI = 0.39) and 12.5 μM (n = 3, CI = 0.14), indicative of potential synergism between the NR5A2-GATA6 axis and BCL-XL expression in regulating proliferation and survival of OAC cells (Fig. 7H–I). Therefore, NR5A2-mediated cancer cell survival may form the basis of potential complementary therapeutic approaches if suitably targeted.

## Discussion

Epidemiological studies have shown that IM and GORD are prominent risk factors in the development of OAC from BO. Thus, accurately defining the intestine-enriched molecular components of BO and their responses to reflux constituents may highlight factors supporting oncogenesis and OAC cell survival. Using data from multiple genomic profiling studies, we have identified a signature of genes differentially expressed between intestinal tissues relative to the oesophageal epithelium. This intestine-enriched signature accurately reflected the functional differences between these tissue types at a gene transcript level. Whole biopsy tissue, which is likely to contain a mixture of cell types, be that epithelial, stromal or immune, was used in our study and in the GEM studies used for meta-profiling. However, the expression profiles generated from these samples proved to be useful in following the BO-OAC sequence suggesting that they are relevant to pathogenesis irrespective of their origin. Additionally, hierarchical clustering of the BO and OAC tissues of these GEM studies demonstrated significant homogeneity of the intestine-like signatures expressed thus supporting the approach. Nonetheless genes which are differentially expressed in intestinal tissue relative to oesophageal tissue are also substantially over-represented in Barrett’s and OAC tissues. Furthermore, the expression of the NR5A2, GATA6 and FOXA2 in the OAC cell lines used in this study also support the epithelial origins of the intestine-like signature.

Our study defined the molecular similarity between the meta-profile of BO and intestinal tissues. Interestingly, the majority (77%) of the genes differentially expressed within the meta-signature of BO were components of the intestine-like signature and, furthermore, were enriched for G-protein (RGS12, RHOC), angiogenic (PDGFA, MCAM) and Wnt signalling regulators (NOTCH2, NR5A2 and GATA6) rather than protein classes representative of intestinal cell digestive function such as exopeptidases, fructose metabolic enzymes and protein transaminases. It is likely that some of the differences observed between these tissue types may be the result of underlying epigenetic differences between intestinal and metaplastic tissues^40^. Nucleic acid binding proteins, as ultimate regulators of cellular identity and transformation, were the main class of proteins represented within the intestine-like signature of BO. NR5A2/LRH1, the most interconnected TF from the gene networking analysis, is an orphan receptor involved in development, bile acid homeostasis, steroidogenesis and has been implicated in the co-ordination of local immune responses and intestinal cell renewal^41^,^42^,^43^,^44^. GATA6 is a zinc finger TF with important roles in organogenesis of the gut, lung and heart, and thus has central roles in regulation of cellular differentiation and proliferation^38^. GATA6 expression has been associated with promotion of cellular invasion and adenoma self-renewal in colon cancer^45^, and with amplification and regulation of Wnt signalling in pancreatic cancers^38^,^39^. Recent evidence has demonstrated that FOXA2 expression may be regulated by *hedgehog* signalling during murine oesophageal embryogenesis, and in BO^17^. The SMAD6 protein is a SMAD1/5/8 inhibitor and immediate-early response gene induced in response to BMP family members to form a regulatory feedback loop. Thus the higher levels of SMAD6 observed in BO in this study likely reflects the ongoing BMP4 signalling as has been previously reported during oesophageal metaplasia^13^,^45^,^46^. It is also likely that other protein classes present within this signature, such as secreted factors and kinases, may play as yet undefined roles. However, these are likely to be mediated through, and regulated by, transcriptional mechanisms and thus reflected by the TFs represented in this study.

The intestine-like signature described in our study and its constituent TFs were again highly expressed in the meta-profile of OAC tissues. Comparatively, a reduced proportion of the non-intestinal signature of BO was expressed upon transition to OAC with loss of TFs such as NR4A3 and ST7 highlighted. ST7 expression has been observed to correlate with cell cycle arrest and abrogate tumourigenicty when expressed in xenograft models resulting in remodelling of tumour microenvironment^47^,^48^. Interestingly, over-expression of NR4A3/NOR1 also inhibited prostate cancer cell growth and reduced the levels of anti-apoptotic proteins BCL2 and BCL-XL^49^. Recent evidence has highlighted that the expression of intestinal factors such as CDX2^35^ and NR5A2^36^ may be down-regulated in colorectal adenocarcinoma. In our study NR5A2 expression was significantly up-regulated in BO, the resulting OAC and in the OAC cell lines. These observations point to molecular differences between the BO-OAC transition and that which occurs from normal intestinal tissue during CRC, and supports the hypothesis that intestinal factors expressed within BO may act in support of oncogenesis. Epigenetic comparisons between normal intestinal tissues and intestinal-type metaplasias may shed further light on the intestinal-enriched pathways utilized by BO in the production of its intestinal phenotype and the resulting cancer risk.

GORD is strongly associated with OAC development, and data from recent GWAS studies have further supported this association^50^,^51^,^52^,^53^,^54^. In our study, the ability of pulsatile low pH exposure, simulating reflux events, to up-regulate the expression of NR5A2 and GATA6 is suggestive of their role in the mechanism promoting metaplasia and neoplasia in conjunction with epigenetic and polymorphic variation. Induction of gene expression was observed to be cumulative, in that repeated exposure to low pH resulted in amplified induction of NR5A2 and GATA6. The greater levels of gene changes observed in response to pulsatile, over that of continuous exposure, may be hypothesized to involve mechanisms such as transcriptional priming or transcriptional stress memory as observed in studies in other model organisms^55^,^56^. These findings do not exclude bile acids from involvement in the development of metaplasia and further studies may be required to examine the relationship between gastric acid, combinations of bile acids and the induction of TFs such as NR5A2 and GATA6. In our study abrogation of NR5A2 function by silencing or antagonism resulted in reduced levels of CDX2 expression, the most widely described intestinal factor associated with intestinal tissue identity. Thus it may be hypothesized that repeated exposure to acid reflux, as observed in GORD, may result in augmented temporal gene regulation during intestinal-type transformation. Nonetheless, once BO is established, abrogation of acid reflux does not substantially impact on the risk of cancer development. Hence, the presence of this intestinal phenotype may in itself be a major contributor to carcinogenesis in the absence of reflux. This hypothesis is supported the by persistence of the IM signature during OAC carcinogenesis, whereas by contrast many intestinal genes appear to be lost during colon cancer. Further studies would be required to investigate this hypothesis and confirm its contributions.

In its traditional role NR5A2 may respond to both extra- and intracellular phospholipids, resulting in DNA binding at LRH-1/NR5A2 responsive elements and transcription of genes involved in cholesterol transport (APOA1, SHP)^41^,^57^,^58^, bile acid metabolism (CYP7A1, CYP8B1)^43^ and gluconeogenesis (GCK, FAS)^58^,^59^. However, recent evidence has demonstrated important roles for NR5A2 in colon and pancreatic cancer postulated to be through hedgehog and Wnt/β-catenin signalling pathways^60^,^61^,^62^,^63^. In our study suppression of NR5A2 function by siRNA-mediated silencing or use of the CPD-3 antagonist, both resulted in significant growth arrest and detectable caspase3-mediated cleavage in OAC cells. In experiments to examine the impact of NR5A2 loss on NR5A2-mediated gene regulation it was discovered that GATA6 levels were greatly affected. GATA6 has been shown to regulate survival in pancreatic cancer and OAC cells and to be important for intestinal stem cell renewal^39^,^45^,^64^. Thus, NR5A2-regulated cellular survival may be facilitated through augmentation of GATA6 levels. This was further supported by the ability of the NR5A2 antagonist to suppress low pH-induced GATA6 expression, and the rescue of antagonist-induced growth arrest by over-expression of GATA6 in OAC cells.

During validation of NR5A2 and GATA6 silencing we demonstrated that greater levels of cell death were nullified by an upregulation of the anti-apoptotic factor BCL-XL. Synergistic effects on OAC cell survival was for the first time demonstrated through combined targeting of either NR5A2, or GATA6 function, and BCL-XL expression. This approach was also utilized to further increase the sensitivity of OAC cell lines to cisplatin and the photo-dynamic therapeutic (PDT) Foscan. Development of chemotherapeutic resistance is observed in many cancer types and especially in OAC, with neo-adjuvant chemotherapy the primary therapy of choice. PDT had been examined for use against oesophageal lesions, however the observed off–target effects have limited their systematic use^65^. Further *in-vivo* model studies are necessary to delineate and confirm the efficacy of these co-targeting approaches. However, the findings support the role of these TFs as components of the intestine-like program present within BO and their signalling in promotion of OAC cell survival.

Recent genome wide association studies have been performed in BO and OAC patient cohorts^53^,^54^. In these studies, 26 SNPs were noted within 1 MB of NR5A2 coding region at a significance cut-off of p < 2 × 10^−5^ in UK evaluation BO dataset. SNP rs1325190 (p = 3E^−5^, OR 1.07) may be functionally significant due to its location within an enhancer region (H3K27Ac mark and digital DNase1 hypersensitivity cluster) upstream of the NR5A2 promoter and within multiple TF binding sites for RELA, RUNX3, CEBPB and FOXM1 (Supplementary Fig. S2 and Supplementary Table S2). SNP rs4800353 located 100 kb from the GATA6 coding region achieved significance (p = 2.69E^−7^, OR = 0.82) when both BO and OAC samples were combined in Supplementary information from the BEACON consortium^53^. This SNP lies within a KRAB (kruppel-associated box) domain containing TFs involved in scaffolding of epigenetic machinery. It may be hypothesized that SNPs capable of altering promoter resident TF binding sites would impact upon expression and regulation of factors such as NR5A2 and GATA6 alone, and in response to GORD.

In summary, we have utilized a meta-analysis approach combining gene expression data sets of OAC carcinogenesis, published GWAS data and functional studies to delineate the role of intestine-enriched factors expressed within BO in the promotion and progression of OAC. A range of mutations in both oncogenes and tumour suppressor have been identified in the Barrett’s cancer sequence. Further pathological and experimental studies may be needed to examine the collaboration between the intestinal factors defined in this study and such genomic and mutational changes arising in cancer.

## Methods

### Sample collection and RNA extraction

Biopsies of similar size and weight were obtained from histologically normal oesophageal (n = 3), duodenal (n = 3) and colonic biopsies (n = 3) from each tissue site from 6 patients (mean age 60.5, 4 male and 2 female) undergoing routine endoscopy for suspected but unfounded gastrointestinal symptoms. Biopsies were taken from 17 BO patients (confirmed for secretory intestinal metaplasia by the presence of goblet cells), 23 pre-treatment OAC patients (with intestinal-metaplasia BO history) and an additional cohort of 17 control patients with no GORD symptoms or BO pathology undergoing routine surveillance for unfounded lower gastrointestinal symptoms such as celiac disease (Supplementary Table S3). The BO patients selected in our study were enrolled in routine follow-up surveillance for BO and thus those with BO of sufficient length could be identified, leading to accuracy in biopsy sampling of only the metaplastic segment. To limit the presence of immune cell types present from potential tissue damage during biopsy procedure, samples were first washed in RNAlater^®^ (Ambion). Samples were stored at 4 °C in RNAlater^®^ for 24 hs prior to storage at −40 °C. In all samples, RNA was extracted by homogenization in TRI-reagent (Sigma) followed by further RNA spin column clean-up (Min Elute, Qiagen). RNA quantitation and qualitation was determined by nano-drop photometer and Bioanalyser RNA module (Agilent), using RIN values >8.0. Written, oral and informed consent was given by all patients who contributed to this study. Ethical approval for this study was granted by the “Research Ethics Committee of Tallaght Hospital, Dublin 24 and St. James’s Hospital, James’s Street, Dublin 8” and carried out in accordance with the associated guidelines.

### Gene-expression microarray (GEM) data analysis and inter-array comparison

Conversion and hybridization of cRNA was performed by Almac Group Sciences (Craigavon, UK) and cRNA samples were hybridized to Affymetrix HGU133A plus 2.0 gene chips. Informatic and statistical analysis was performed using Genespring™ (Agilent). Original. cel. chp files for normal gastro-intestinal tissues are available at the gene expression omnibus (GSE40220). Initially, typical baseline intestinal gene expression was defined by Mann-Whitney testing between oesophageal, colonic and duodenal tissues (FC > 1.5, p < 0.001). Gene expression microarray study data of BO and OAC patient biopsies by Kimchi *et al.* (GSE1420)^31^, Ostrowski *et al.* (GSE36223)^32^, Silvers *et al.* (GSE37203)^34^ and Wang *et al.* (GSE26886)^33^ were acquired from the gene expression omnibus and re-analysed in Genespring™ (Agilent) at Mann-Whitney test p < 0.001 and FC ± 1.5, using HGU133A annotation data mask for generation of shared BO and OAC meta-profiles (Supplementary Table S4). Other gastrointestinal and non-gastrointestinal GEM studies were similarly analysed and are listed in S4 Table. Further methods available in online Supplementary methods.

### Real-time RT-PCR and gene expression analysis

Total RNA was transcribed to complementary DNA using the RETROscript RT–PCR components as previously described (Ambion)^66^. The PCR reactions were prepared in 384-well format and used in the ABI Prism 7900HT thermocycler (Applied Biosystems). Relative fold-change was calculated using the ΔΔCt method with glyceraldehyde 3-phosphate dehydrogenase (GAPDH) as the denominator and using either untreated control resting cell lines or mean values from normal control oesophageal tissue cohort for comparison of gene expression in disease tissue or treated samples. Two-tailed Non-parametric Mann-Whitney testing suitable for real time gene expression data was performed in Graphpad Prism™ to determine significance.

### Simulated reflux experiments

Oesophageal cell lines HET-1A, SKGT4 and FLO1 were obtained from ATCC, cultured as previously described and routinely authenticated by STR profiling^66^. Cells were routinely cultured at sub-confluent levels in RPMI media (Gibco) containing sodium bicarbonate (standard media) and daily media changes to limit fluctuations in pH, monitored by sterile pH monitor. Plates were seeded at 5 × 10^5^ (6-well) or 3 × 10^4^ (96-well, viability) cells per well, in RPMI buffered with sodium bicarbonate, 24 hs prior to start of acidic exposure experiments. Exposure of cells to gastric acid was simulated by replacement of the culture media with acidified base RPMI media (without additives or sodium bicarbonate buffer) titrated to pH4.5 with hydrochloric acid and used immediately for 10 min (pulsatile) or in continuous conditions. Control cells were treated with fresh unbuffered RPMI at pH7.4 for 10 min or continuously. Cell culture pH was measured and RNA samples preserved at the following time-points prior to, and during, the experiment:- t-2, t0, t+2, t+4 and t+24 hs. Acidic exposure was achieved as follows:- at t0 hs media was removed and replaced with acidified (pH4.5) or standard non-acidified (pH7.4) RPMI media, without sodium bicarbonate, for 10 min or 24 hs (continuous exposure) followed by a return to buffered media at pH7.4; at t+2 hs cells were once again exposed to acidified (pH4.5) or non-acidified (pH7.4) RPMI media for 10 mins followed again by return to buffered media at pH7.4 for a further 2 hs (t+4 hs); this 10 min pulse was once again performed and samples were taken at t+24 hs. In viability experiments the same pulsing procedure was utilized but additional measurements were taken at 8 hs to demonstrate the decline in cell viability in continuous exposure to pH4.5. Similarly protein samples were taken at additional time points to that of RNA experiments to reflect the latent increases at the protein level that follow changes at mRNA level. These experiments were performed with 3 independent biological replicate and 3 technical replicate experiments and statistical significance applied by two-tailed non-parametric t-test suitable for gene expression analysis or students t-test in viability experiments (Graphpad Prism™).

### Antagonist treatment, siRNA-mediated gene silencing and cell death assays

NR5A2 antagonist CPD-3 (1-(3′-1-[2-(4-morpholinyl)ethyl]-1*H*-pyrazol-3-yl-3-biphenylyl)ethanone), as first developed by Benod *et al.*^67^, was custom synthesized (Chembridge) and dissolved in 0.1% DMSO, as the vehicle. SiRNA smartpools™, of 4 independent siRNA sequences which reduce off-target effects, for the non-targeting control (siNT), positive cell death control (siTOX), NR5A2 (siNR5A2), GATA6 (siGATA6) and BCL-XL (siBCL-XL) were transfected using Dharmafect 4 (GE Dharmacon). Combined inhibition of GATA6 and BCL-XL was achieved through direct mixing of equimolar solutions of the respective siRNAs in siRNA buffer. Inhibition of target gene expression by siRNA transfection was verified by RT-PCR and Western blotting utilizing NR5A2 (ab41901, Abcam), GATA6 (A549, Cell Signaling), BCL-XL (2762S, Cell Signaling) and Histone H3 (9715, Cell Signaling) antibodies. Chemiluminescent Western blots for GATA6 experiments were imaged by zoomed/cropped live image capture by LAS-4000 CCD imager (Fujifilm Life Science/GE). Western blots in NR5A2 and BCL-XL experiments were developed using exposure to traditional X-ray film and development protocols (expanded blots available in Fig. S3). Over-expression pCMV-entry GATA6 clone (PS100001) and empty vectors (Origene) were transfected in SKGT4 cells using lipofectamine 2000 (Invitrogen). Cisplatin (P4394, Sigma) was dissolved in sterile water (Sigma) and used at concentrations indicated. Photo-mediated cell death was achieved through exposure of cell lines to PDT agent temopofrin (Foscan) for 24 hs in the dark prior to exposure to light at 633 nm as further detailed in Vaz G *et al.*^68^. Cell viability and caspase-3/7 activation was determined following gene silencing by MTT assay (LGC Prochem) and CaspaseGLO luminescent caspase-3/7 cleavage assay (Promega), and significance determined by unpaired students t-test (Graphpad Prism). Synergism and CI score for non-constant ratio was analysed determined by Chou-Talalay testing (Compusyn™ software)^69^.

## Additional Information

**How to cite this article**: Duggan, S. P. *et al.* The characterization of an intestine-like genomic signature maintained during Barrett's-associated adenocarcinogenesis reveals an NR5A2-mediated promotion of cancer cell survival. *Sci. Rep.* **6**, 32638; doi: 10.1038/srep32638 (2016).

## Supplementary Material

Supplementary Information

## References

[b1] Jung K. W. *et al.* Epidemiology and natural history of intestinal metaplasia of the gastroesophageal junction and Barrett’s esophagus: a population-based study. The American Journal of Gastroenterology 106, 1447, doi: 10.1038/ajg.2011.130 (2011). 21483461 PMC3150349

[b2] El-Serag H. B., Sweet S., Winchester C. C. & Dent J. Update on the epidemiology of gastro-oesophageal reflux disease: a systematic review. Gut 63, 871–880, doi: 10.1136/gutjnl-2012-304269 (2013). 23853213 PMC4046948

[b3] von Holzen U. & Enders G. H. A surprise cell of origin for Barrett’s esophagus. Cancer Biology & Therapy 13, 588–591, doi: 10.4161/cbt.20088 (2012). 22549156 PMC3408968

[b4] Fitzgerald R. C. *et al.* British Society of Gastroenterology guidelines on the diagnosis and management of Barrett’s oesophagus. Gut 63, 7–42, doi: 10.1136/gutjnl-2013-305372 (2013). 24165758

[b5] Cook M. B. *et al.* Gastroesophageal reflux in relation to adenocarcinomas of the esophagus: a pooled analysis from the Barrett’s and Esophageal Adenocarcinoma Consortium (BEACON). Plos ONE 9, e103508, doi: 10.1371/journal.pone.0103508 (2014). 25075959 PMC4116205

[b6] Krishnamoorthi R. *et al.* Rates and predictors of progression to esophageal carcinoma in a large population-based Barrett’s esophagus cohort. Gastrointestinal endoscopy, doi: 10.1016/j.gie.2015.12.036 (2016). PMC4912845 26772891

[b7] de Jonge P. J. F. *et al.* Risk of malignant progression in patients with Barrett’s oesophagus: a Dutch nationwide cohort study. Gut 59, 1030–1036, doi: 10.1136/gut.2009.176701 (2010). 20639249

[b8] Sikkema M., de Jonge P. J. F., Steyerberg E. W. & Kuipers E. J. Risk of esophageal adenocarcinoma and mortality in patients with Barrett’s esophagus: a systematic review and meta-analysis. Clinical gastroenterology and hepatology : the official clinical practice journal of the American Gastroenterological Association 8, doi: 10.1016/j.cgh.2009.10.010 (2009). 19850156

[b9] Bhat S. *et al.* Risk of malignant progression in Barrett’s esophagus patients: results from a large population-based study. JNCI Journal of the National Cancer Institute 103, 1049–1057, doi: 10.1093/jnci/djr203 (2011). 21680910 PMC3632011

[b10] Nicholson A. M. *et al.* Barrett’s metaplasia glands are clonal, contain multiple stem cells and share a common squamous progenitor. Gut 61, 1380–1389, doi: 10.1136/gutjnl-2011-301174 (2011). 22200839

[b11] Singh S., Garg S. K., Singh P. P., Iyer P. G. & El-Serag H. B. Acid-suppressive medications and risk of oesophageal adenocarcinoma in patients with Barrett’s oesophagus: a systematic review and meta-analysis. Gut 63, 1229–1237, doi: 10.1136/gutjnl-2013-305997 (2013). 24221456 PMC4199831

[b12] Wang X. *et al.* Residual embryonic cells as precursors of a Barrett’s-like metaplasia. Cell 145, 1023–1035, doi: 10.1016/j.cell.2011.05.026 (2011). 21703447 PMC3125107

[b13] Barros R. *et al.* Key elements of the BMP/SMAD pathway co-localize with CDX2 in intestinal metaplasia and regulate CDX2 expression in human gastric cell lines. The Journal of Pathology 215, 411–420, doi: 10.1002/path.2369 (2008). 18498120

[b14] Kong J., Crissey M. A., Funakoshi S., Kreindler J. L. & Lynch J. P. Ectopic Cdx2 expression in murine esophagus models an intermediate stage in the emergence of Barrett’s esophagus. Plos ONE 6, e18280, doi: 10.1371/journal.pone.0018280 (2011). 21494671 PMC3071814

[b15] Mari L. *et al.* A pSMAD/CDX2 complex is essential for the intestinalization of epithelial metaplasia. Cell reports 7, 1197–1210, doi: 10.1016/j.celrep.2014.03.074 (2014). 24794431

[b16] Hwang J. T. K. & Kelly G. M. GATA6 and FOXA2 regulate Wnt6 expression during extraembryonic endoderm formation. Stem Cells and Development 21, 3220–3232, doi: 10.1089/scd.2011.0492 (2012). 22607194

[b17] Wang D. H. *et al.* Hedgehog signaling regulates FOXA2 in esophageal embryogenesis and Barrett’s metaplasia. Journal of Clinical Investigation 124, 3767–3780, doi: 10.1172/JCI66603 (2014). 25083987 PMC4151220

[b18] Watts J. A. *et al.* Study of FoxA pioneer factor at silent genes reveals Rfx-repressed enhancer at Cdx2 and a potential indicator of esophageal adenocarcinoma development. Plos Genet 7, e1002277, doi: 10.1371/journal.pgen.1002277 (2011). 21935353 PMC3174211

[b19] Wong N. A. C. S. *et al.* CDX1 is an important molecular mediator of Barrett’s metaplasia. Proceedings of the National Academy of Sciences 102, 7565–7570, doi: 10.1073/pnas.0502031102 (2005). PMC1140438 15894614

[b20] Kazumori H., Ishihara S. & Kinoshita Y. Roles of caudal-related homeobox gene Cdx1 in oesophageal epithelial cells in Barrett’s epithelium development. Gut 58, 620–628, doi: 10.1136/gut.2008.152975 (2009). 19136512

[b21] Xie Y. *et al.* Overexpression of Cdx2 inhibits progression of gastric cancer *in vitro*. International Journal of Oncology 36, 509–516 (2010). 20043087

[b22] Watari J. *et al.* Differences in genetic instability and cellular phenotype among Barrett’s, cardiac, and gastric intestinal metaplasia in a Japanese population with Helicobacter pylori. Histopathology 55, 261–269, doi: 10.1111/j.1365-2559.2009.03370.x (2009). 19723140 PMC4458565

[b23] Bandla S. *et al.* Comparison of cancer-associated genetic abnormalities in columnar-lined esophagus tissues with and without goblet cells. Annals of Surgery 260, 72–80, doi: 10.1097/SLA.0000000000000424 (2014). 24509200 PMC4047149

[b24] Salemme M. *et al.* Intestinal metaplasia in Barrett’s oesophagus: An essential factor to predict the risk of dysplasia and cancer development. Dig Liver Dis, doi: 10.1016/j.dld.2015.10.021 (2015). 26614646

[b25] Chandrasoma P. *et al.* Columnar-lined esophagus without intestinal metaplasia has no proven risk of adenocarcinoma. The American Journal of Surgical Pathology 36, 1–7, doi: 10.1097/PAS.0b013e31822a5a2c (2011). 21959311

[b26] Lind A. *et al.* The immune cell composition in Barrett’s metaplastic tissue resembles that in normal duodenal tissue. Plos ONE 7, e33899, doi: 10.1371/journal.pone.0033899 (2012). 22509265 PMC3317926

[b27] Arul G. S. *et al.* Mucin gene expression in Barrett’s oesophagus: an *in situ* hybridisation and immunohistochemical study. Gut 47, 753–761, doi: 10.1136/gut.47.6.753 (2000). 11076872 PMC1728131

[b28] Corfield A. P. *et al.* Mucins and mucosal protection in the gastrointestinal tract: new prospects for mucins in the pathology of gastrointestinal disease. Gut 47, 589–594, doi: 10.1136/gut.47.4.589 (2000). 10986224 PMC1728059

[b29] Sawhney R. A. *et al.* Morphological characterization of the squamocolumnar junction of the esophagus in patients with and without Barrett’s epithelium. Digestive diseases and sciences 41, 1088–1098 (1996). 8654139 10.1007/BF02088224

[b30] Fléjou J.-F. Barrett’s oesophagus: from metaplasia to dysplasia and cancer. Gut 54 Suppl 1, i6–12, doi: 10.1136/gut.2004.041525 (2005). 15711008 PMC1867794

[b31] Kimchi E. T. *et al.* Progression of Barrett’s metaplasia to adenocarcinoma is associated with the suppression of the transcriptional programs of epidermal differentiation. Cancer research 65, 3146–3154, doi: 10.1158/0008-5472.CAN-04-2490 (2005). 15833844

[b32] Ostrowski J. *et al.* Molecular defense mechanisms of Barrett’s metaplasia estimated by an integrative genomics. Journal of Molecular Medicine 85, 733–743, doi: 10.1007/s00109-007-0176-3 (2007). 17415542

[b33] Wang Q., Ma C. & Kemmner W. Wdr66 is a novel marker for risk stratification and involved in epithelial-mesenchymal transition of esophageal squamous cell carcinoma. BMC cancer 13, 137, doi: 10.1186/1471-2407-13-137 (2013). 23514407 PMC3610187

[b34] Silvers A. L. *et al.* Decreased selenium-binding protein 1 in esophageal adenocarcinoma results from posttranscriptional and epigenetic regulation and affects chemosensitivity. Clinical Cancer Research 16, 2009–2021, doi: 10.1158/1078-0432.CCR-09-2801 (2010). 20332323 PMC2953959

[b35] Dalerba P. *et al.* CDX2 as a Prognostic Biomarker in Stage II and Stage III Colon Cancer. N Engl J Med 374, 211–222, doi: 10.1056/NEJMoa1506597 (2016). 26789870 PMC4784450

[b36] Abu-Remaileh M. *et al.* Chronic inflammation induces a novel epigenetic program that is conserved in intestinal adenomas and in colorectal cancer. Cancer Res 75, 2120–2130, doi: 10.1158/0008-5472.CAN-14-3295 (2015). 25808873

[b37] Gharahkhani P. *et al.* Chronic gastroesophageal reflux disease shares genetic background with esophageal adenocarcinoma and Barrett’s esophagus. Hum Mol Genet, doi: 10.1093/hmg/ddv512 (2015). PMC4743691 26704365

[b38] Beuling E. *et al.* GATA6 is required for proliferation, migration, secretory cell maturation, and gene expression in the mature mouse colon. Molecular and Cellular Biology 32, 3392–3402, doi: 10.1128/MCB.00070-12 (2012). 22733991 PMC3422006

[b39] Lin L. *et al.* Activation of GATA binding protein 6 (GATA6) sustains oncogenic lineage-survival in esophageal adenocarcinoma. Proceedings of the National Academy of Sciences 109, 4251–4256, doi: 10.1073/pnas.1011989109 (2012). PMC3306720 22375031

[b40] Krause L. *et al.* Identification of the CIMP-like subtype and aberrant methylation of members of the chromosomal segregation and spindle assembly pathways in esophageal adenocarcinoma. Carcinogenesis 37, 356–365, doi: 10.1093/carcin/bgw018 (2016). 26905591 PMC4806711

[b41] Fernandez-Marcos P. J., Auwerx J. & Schoonjans K. Emerging actions of the nuclear receptor LRH-1 in the gut. Biochimica et biophysica acta 1812, 947–955, doi: 10.1016/j.bbadis.2010.12.010 (2010). 21194563 PMC3617401

[b42] Heng J.-C. D. *et al.* The nuclear receptor Nr5a2 can replace Oct4 in the reprogramming of murine somatic cells to pluripotent cells. Cell stem cell 6, 167–174, doi: 10.1016/j.stem.2009.12.009 (2010). 20096661

[b43] Out C. *et al.* Liver receptor homolog-1 is critical for adequate up-regulation of Cyp7a1 gene transcription and bile salt synthesis during bile salt sequestration. Hepatology 53, 2075–2085, doi: 10.1002/hep.24286 (2011). 21391220

[b44] Atanasov A. G. *et al.* Cell cycle-dependent regulation of extra-adrenal glucocorticoid synthesis in murine intestinal epithelial cells. The FASEB Journal 22, 4117–4125, doi: 10.1096/fj.08-114157 (2008). 18711026

[b45] Whissell G. *et al.* The transcription factor GATA6 enables self-renewal of colon adenoma stem cells by repressing BMP gene expression. Nature Cell Biology 16, 695–707, doi: 10.1038/ncb2992 (2014). 24952462

[b46] Kestens C., Siersema P. D., Offerhaus G. J. & van Baal J. W. BMP4 Signaling Is Able to Induce an Epithelial-Mesenchymal Transition-Like Phenotype in Barrett’s Esophagus and Esophageal Adenocarcinoma through Induction of SNAIL2. Plos One 11, e0155754, doi: 10.1371/journal.pone.0155754 (2016). 27191723 PMC4871520

[b47] Charong N., Patmasiriwat P. & Zenklusen J. C. Localization and characterization of ST7 in cancer. Journal of Cancer Research and Clinical Oncology 137, 89–97, doi: 10.1007/s00432-010-0863-2 (2010). 20238225 PMC11828306

[b48] Hooi C.-F. *et al.* ST7-mediated suppression of tumorigenicity of prostate cancer cells is characterized by remodeling of the extracellular matrix. Oncogene 25, 3924–3933, doi: 10.1038/sj.onc.1209418 (2006). 16474848

[b49] Shan Z. *et al.* Overexpression of oxidored-nitro domain containing protein 1 induces growth inhibition and apoptosis in human prostate cancer PC3 cells. Oncology Reports 32, 1939–1946, doi: 10.3892/or.2014.3407 (2014). 25118646

[b50] Cook M. B. *et al.* Gastroesophageal reflux in relation to adenocarcinomas of the esophagus: a pooled analysis from the Barrett’s and Esophageal Adenocarcinoma Consortium (BEACON). Plos One 9, e103508, doi: 10.1371/journal.pone.0103508 (2014). 25075959 PMC4116205

[b51] Dai J. Y. *et al.* A newly identified susceptibility locus near FOXP1 modifies the association of gastroesophageal reflux with Barrett’s esophagus. Cancer Epidemiol Biomarkers Prev 24, 1739–1747, doi: 10.1158/1055-9965.EPI-15-0507 (2015). 26377193 PMC4816532

[b52] Gharahkhani P. *et al.* Chronic gastroesophageal reflux disease shares genetic background with esophageal adenocarcinoma and Barrett’s esophagus. Hum Mol Genet 25, 828–835, doi: 10.1093/hmg/ddv512 (2016). 26704365 PMC4743691

[b53] Levine D. M. *et al.* A genome-wide association study identifies new susceptibility loci for esophageal adenocarcinoma and Barrett’s esophagus. Nat Genet 45, 1487–1493, doi: 10.1038/ng.2796 (2013). 24121790 PMC3840115

[b54] Su Z. *et al.* Common variants at the MHC locus and at chromosome 16q24.1 predispose to Barrett’s esophagus. Nature Genetics 44, 1131–1136, doi: 10.1038/ng.2408 (2012). 22961001 PMC3459818

[b55] Lin Y., Sohn C. H., Dalal C. K., Cai L. & Elowitz M. B. Combinatorial gene regulation by modulation of relative pulse timing. Nature 527, 54–58, doi: 10.1038/nature15710 (2015). 26466562 PMC4870307

[b56] Sailaja B. S., Cohen-Carmon D., Zimmerman G., Soreq H. & Meshorer E. Stress-induced epigenetic transcriptional memory of acetylcholinesterase by HDAC4. Proc Natl Acad Sci USA 109, E3687–E3695, doi: 10.1073/pnas.1209990110 (2012). 23236169 PMC3535662

[b57] Botrugno O. A. *et al.* Synergy between LRH-1 and beta-catenin induces G1 cyclin-mediated cell proliferation. Molecular cell 15, 499–509, doi: 10.1016/j.molcel.2004.07.009 (2004). 15327767

[b58] Oosterveer M. H. *et al.* LRH-1-dependent glucose sensing determines intermediary metabolism in liver. J Clin Invest 122, 2817–2826, doi: 10.1172/JCI62368 (2012). 22772466 PMC3408738

[b59] Oosterveer M. H. & Schoonjans K. Hepatic glucose sensing and integrative pathways in the liver. Cell Mol Life Sci 71, 1453–1467, doi: 10.1007/s00018-013-1505-z (2014). 24196749 PMC11114046

[b60] Benod C. *et al.* Nuclear receptor liver receptor homologue 1 (LRH-1) regulates pancreatic cancer cell growth and proliferation. Proc Natl Acad Sci USA 108, 16927–16931, doi: 10.1073/pnas.1112047108 (2011). 21949357 PMC3193228

[b61] Bianco S., Jangal M., Garneau D. & Gevry N. LRH-1 controls proliferation in breast tumor cells by regulating CDKN1A gene expression. Oncogene 34, 4509–4518, doi: 10.1038/onc.2014.382 (2015). 25435372

[b62] Kramer H. B. *et al.* LRH-1 drives colon cancer cell growth by repressing the expression of the CDKN1A gene in a p53-dependent manner. Nucleic Acids Res 44, 582–594, doi: 10.1093/nar/gkv948 (2016). 26400164 PMC4737183

[b63] Wagner R. T., Xu X., Yi F., Merrill B. J. & Cooney A. J. Canonical Wnt/beta-catenin regulation of liver receptor homolog-1 mediates pluripotency gene expression. Stem Cells 28, 1794–1804, doi: 10.1002/stem.502 (2010). 20734354 PMC2996860

[b64] Kwei K. A. *et al.* Genomic profiling identifies GATA6 as a candidate oncogene amplified in pancreatobiliary cancer. PLoS Genet 4, e1000081, doi: 10.1371/journal.pgen.1000081 (2008). 18535672 PMC2413204

[b65] Fayter D., Corbett M., Heirs M., Fox D. & Eastwood A. A systematic review of photodynamic therapy in the treatment of pre-cancerous skin conditions, Barrett’s oesophagus and cancers of the biliary tract, brain, head and neck, lung, oesophagus and skin. Health Technology Assessment 14, 1–288, doi: 10.3310/hta14370 (2010). 20663420

[b66] Duggan S. P. *et al.* An integrative genomic approach in oesophageal cells identifies TRB3 as a bile acid responsive gene, downregulated in Barrett’s oesophagus, which regulates NF-kappaB activation and cytokine levels. Carcinogenesis 31, 936–945, doi: 10.1093/carcin/bgq036 (2010). 20139130

[b67] Benod C. *et al.* Structure-based discovery of antagonists of nuclear receptor LRH-1. Journal of Biological Chemistry 288, 19830–19844, doi: 10.1074/jbc.M112.411686 (2013). 23667258 PMC3707686

[b68] Vaz G. M., Paszko E., Davies A. M. & Senge M. O. High content screening as high quality assay for biological evaluation of photosensitizers *in vitro*. Plos One 8, e70653, doi: 10.1371/journal.pone.0070653 (2013). 23923014 PMC3726630

[b69] Chou T. C., Motzer R. J., Tong Y. Z. & Bosl G. J. Computerized quantitation of synergism and antagonism of taxol, topotecan, and cisplatin against human teratocarcinoma cell-growth - A rational approach to clinical protocol design. JNCI Journal of the National Cancer Institute 86, 1517–1524, doi: 10.1093/jnci/86.20.1517 (1994). 7932806

