# Corrigendum: The characterization of an intestine-like genomic signature maintained during Barrett’s-associated adenocarcinogenesis reveals an NR5A2-mediated promotion of cancer cell survival

**DOI:** 10.1038/srep35330

**Published:** 2016-10-14

**Authors:** Shane P. Duggan, Fiona M. Behan, Murat Kirca, Abdul Zaheer, Sarah A. McGarrigle, John V. Reynolds, Gisela M. F. Vaz, Mathias O. Senge, Dermot Kelleher

Scientific Reports 6: Article number: 3263810.1038/srep32638; published online: 09 02 2016; updated: 10 14 2016

The Acknowledgements section in this Article is incomplete.

“We thank Xinzhong Li and Anthony Davies for support”.

should read

“We thank Xinzhong Li and Anthony Davies for technical support. We would also like to acknowledge funding support from the Health Research Board Ireland (HRB-TRA.2007.11 and the HRB PhD Scholars programme) and the University of British Columbia Start-up funds F15-04616”.

