# Supplementary material for: The characterization of an intestine-like genomic signature maintained during Barrett’s-associated adenocarcinogenesis reveals an NR5A2-mediated promotion of cancer cell survival
Source: Sci Rep. 2016 Sep 2;6:32638. doi: 10.1038/srep32638 (PMC5009315; doi:10.1038/srep32638)
Supplement: Supplementary Information [file srep32638-s1.pdf]

The characterization of an intestine-like genomic signature maintained during Barrett's-associated adenocarcinogenesis reveals an NR5A2-mediated promotion of cancer cell survival.

Shane P Duggan[1,2*], Fiona M Behan[3], Murat Kirca[4], Abdul Zaheer[4], Sarah A McGarrigle[5], John V Reynolds[5], Gisela M.F. Vaz[6], Mathias O Senge[6], Dermot Kelleher[1,2].

Affiliations:-

[1]Division of Gastroenterology, University of British Columbia, 2775 Laurel Street, Vancouver, British Columbia, Canada.

[2]Life Science Institute, 2350 Health Sciences Mall, Vancouver, British Columbia, Canada.

[3]Department of Clinical Medicine, Institute of Molecular Medicine, Trinity College Dublin, St James' Hospital, Dublin, Ireland

[4]Department of Gastroenterology, St James' Hospital, Dublin, Ireland.

[5]Department of Surgery, Institute of Molecular Medicine, Trinity College Dublin, St James' Hospital, Dublin 8, Ireland

[6]Medicinal Chemistry, Trinity Translational Medicine Institute, Trinity College Dublin, the University of Dublin, St James' Hospital, Dublin 8, Ireland.

[*]Corresponding author: shane.duggan@ubc.ca

Corresponding Author:

Dr Shane Duggan, Rm 5.408, [2]Life Science Institute, 2350 Health Sciences Mall, Vancouver, British Columbia, Canada, BC V6T 1Z3, Canada. Tel; 001 6048225988, email: shane.duggan@ubc.ca

Supplementary Figures and Tables:

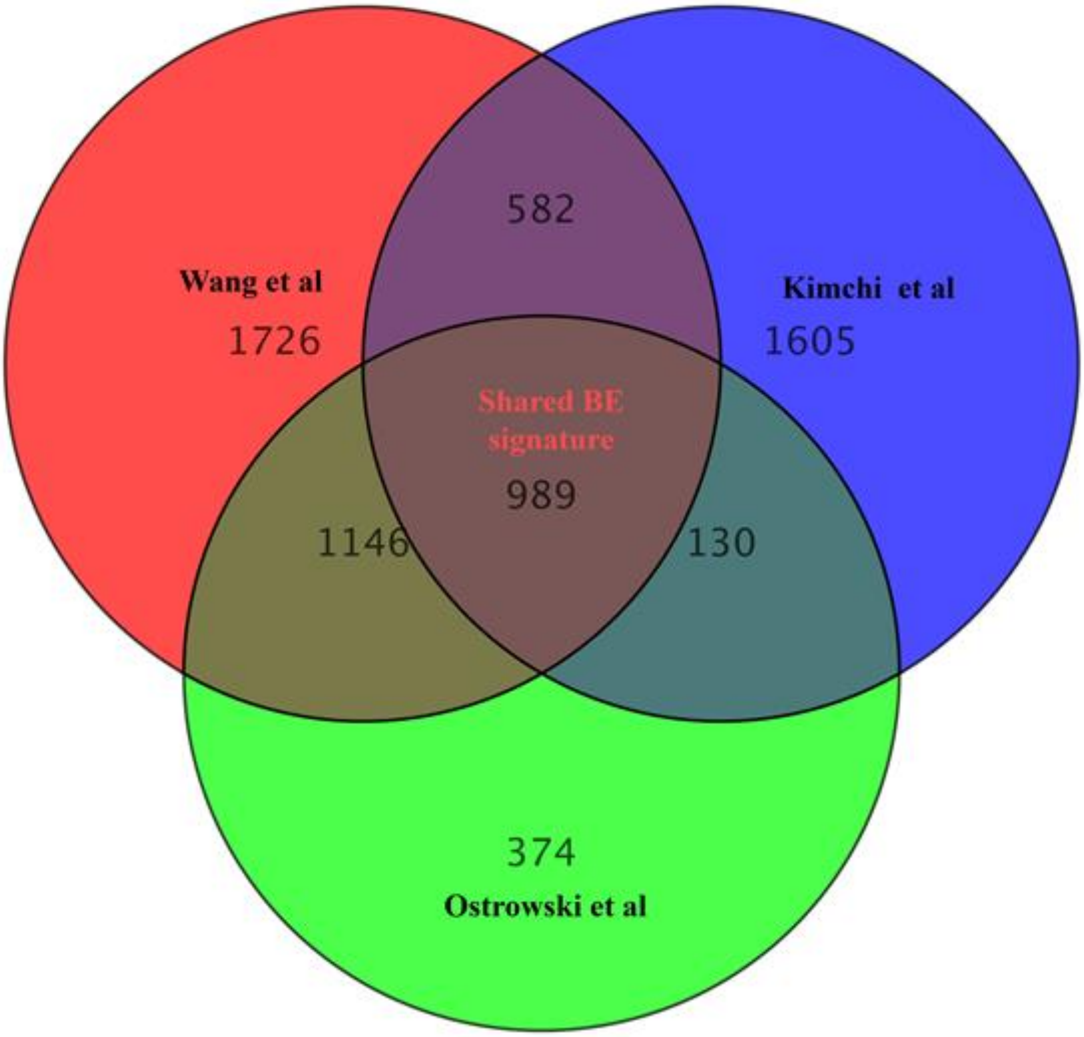

Figure S1: The shared signature of BO as determined through analysis of the BO signature in 3 gene expression microarray studies. All studies were examined at the same cut off (p<0.001, Mann-Whitney non-parametric testing, fold change>1.5) using normal squamous tissue within each study as control. The combined signature was then derived by Venn diagram between the resulting gene lists.

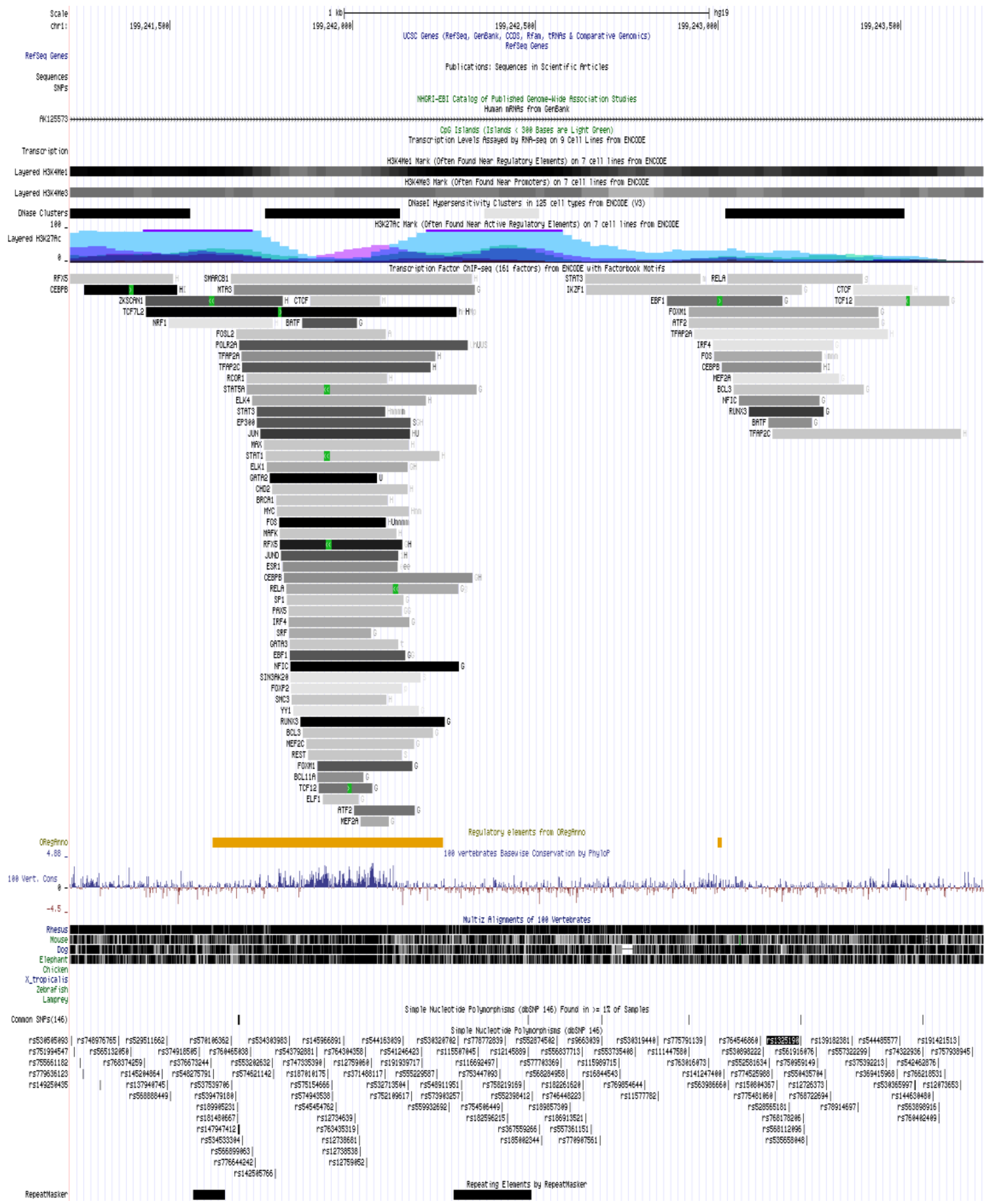

Figure S2: Single nucleotide polymorphism rs1325190 located within an enhancer region upstream of the NR5A2 promoter. The enhancer region is marked by both digital DNase1 hypersensitivity cluster and a H3K27Ac marker. Rs1325190 resides within a transcription factor binding site (TFBS) cluster close to further significant cluster of TFBSs.

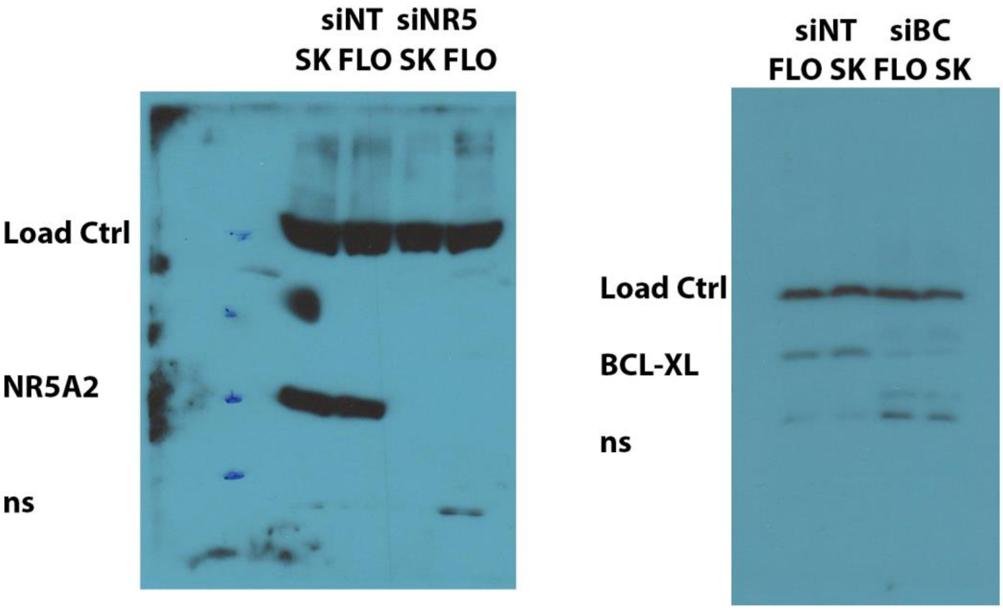

Figure S3:- Expansion of traditional X-ray film based Western blots for cropped images from figure 6A and figure 7D. Western blotting chemiluminescent light was performed as per material and methods and captured both both traditional X-ray film capture (as above) and by live zoomed/cropped imaging of band regions by digital CCD camera (no full scan of entire blot obtainable). Load Ctrl:- protein loading controls; ns:- non-specific bands.

Table S1:- Intestinal signature in BO, OAC and OSCC gene expression microarray studies, Proportion of gene profile of each lesion associated with the intestinal signature as percentage, as determined by Mann-Whiney testing at $p<0.001$ and FC>1.5 between disease and control tissues.

GEM study	INT in BE	INT in EAC	INT in ESCC
Kimchi *et al*	51%	39.40%	
Wang *et al*	57%	49.10%	13.20%
Ostrowski *et al*	67.90%	na	
Average (above)	58.30%	44.25%	
Shared Profile	77.70%	60.50%	

Table S2: HaploReg results for rs1325190 detailing linkage, population frequencies Enhancer makers, bound factors and motifs altered.

chr	pos (hg19)	LD (r^2)	LD (D')	variant	Ref	Alt	AFR freq	AMR freq	ASN freq	EUR freq	SiPhy cons	Promoter histone marks	Enhancer histone marks	DNAse	Proteins bound	eQTL tissues	Motifs changed
1	199243228	1	1	rs1325190	G	A	0.09	0.3	0.33	0.27			7 cell types	41 cell types	MEF2A,CEBPB		4 altered motifs
1	199243829	1	1	rs4570445	T	C	0.1	0.3	0.33	0.27							
1	199244189	0.99	1	rs1325191	T	C	0.08	0.31	0.33	0.27							

Table S3: Patient Cohort characteristics and demographics for real-time RT_PCR and gene expression microarray analysis.

	n	Mean Age	Age Range
Patients	68		
Normal oesophagus	20	61.3	18-78
Normal duodenum	3	64	63-65
Normal Colon	3	63.3	63-64
Barrett's Metaplasia (Goblet cell positive)	17	63.3	37-83
Length of BE lesion <3	2		
Length of BE lesion >3	15		
Dysplasia	2	57.5	55-60
Oesophageal adenocarcinoma	23	64	45-82

Table S4: Gene expression microarray studies of normal and cancer tissues utilized.

GEO Accession	Tissue Types	Platform	Study Authors
GSE1420	SQ BE EAC	Affy-GLP96	Kimchi et al
GSE36223	SQ BE EAC	Affy-GLP96	Ostrowski et al
GSE37203	BE EAC	Affy-GLP96	Silvers et al
GSE26886	SQ BE EAC ESCC	Affy-GLP96	Wang et al
GSE41258	NC TU	Affy-GLP96	Sheffer et al
GSE2503	NS SCC	Affy-GLP96	Nindl et al
GSE20437	NBE BC	Affy-GLP96	

SQ-squamous; BE- Barrett's esophagu; EAC-esophageal adenocarcinoma; ESCC- Esophageal squamous cell carcinoma

NC-Normal colon; TU-Tumour; NS- Normal skin; SCC- Squamous cell carcinoma; NBE- Normal Breast epithelium; BC- Breast cancer

Supplementary methods:

Gene expression microarray analyses and informatics

Conversion and hybridization of cRNA was performed by Almac Group Sciences (Craigavon, UK) and cRNA samples were hybridized to Affymetrix HGU133A plus 2.0 gene chips. Informatic and statistical analysis was performed using Genespring™ (Agilent). Data acquisition and .cel file generation was performed by GCOS followed by normalization by robust multichip average (RMA) in Genespring™ software. Original .cel .chp files for normal gastro-intestinal tissues are available at the gene expression omnibus (GSE40220). Gene expression microarray study data of BE and EAC patient biopsies by Kimchi *et al* (GSE1420),[1] Ostrowski *et al* (GSE36223),[2] Silvers *et al* (GSE37203)[3] and Wang et al (GSE26886)[4] were acquired from the gene expression omnibus and utilized to examine the intestinal signature of BE and

EAC (Supplementary Table 4). Initially, typical baseline intestinal gene expression was defined by Mann-Whitney inter-comparisons between oesophageal, colonic and duodenal tissues (FC>1.5, Mann-Whitney p<0.001) generating a shared intestinal profile versus the oesophageal tissue. Venn diagram comparisons were then utilized to inter-compare this intestinal gene list with the differential gene signature of BE and EAC also defined at FC>1.5 and p<0.001 in non-parametric testing (Mann-Whitney, Supplementary Table 1). The proportion of this signature expressed by other cancer types was defined similarly and displayed as a percentage of total genes within each study at the same statistical and fold change cut-off. Other gastrointestinal and non-gastrointestinal GEM studies utilized are listed in supplementary Table 4 (S4 Table). Statistical re-analysis of these datasets was performed using HGU133A annotation data mask to provide a common platform of gene/probe numbers and a common statistical approach (Non-parametric Mann-Whitney testing at p<0.001, between controls and disease tissue all with fold change cut-off at FC=1.5) to interpretation between all platforms. Gene networking model interpretation of the data was performed using Ingenuity Pathway Analysis (IPA) (www.ingenuity.com, Redwood City, CA, USA). Further tools used for gene annotation, classification and functional enrichment were Database for Annotation, Visualization and Integrated Discovery (DAVID)[5] and (Protein ANalysis THrough Evolutionary Relationships) PANTHERdb.[6, 7]

1. Kimchi ET, Posner MC, Park JO, Darga TE, Kocherginsky M, Karrison T, et al. Progression of Barrett's metaplasia to adenocarcinoma is associated with the suppression of the transcriptional programs of epidermal differentiation. Cancer research. 2005;65(8):3146-54. doi: 10.1158/0008-5472.CAN-04-2490.
2. Ostrowski J, Mikula M, Karczmarski J, Rubel T, Wyrwicz LS, Bragoszewski P, et al. Molecular defense mechanisms of Barrett's metaplasia estimated by an integrative genomics. Journal of Molecular Medicine. 2007;85(7):733-43. doi: 10.1007/s00109-007-0176-3.
3. Silvers AL, Lin L, Bass AJ, Chen G, Wang Z, Thomas DG, et al. Decreased selenium-binding protein 1 in esophageal adenocarcinoma results from posttranscriptional and epigenetic regulation and affects chemosensitivity. Clinical Cancer Research. 2010;16(7):2009-21. doi: 10.1158/1078-0432.CCR-09-2801.
4. Wang Q, Ma C, Kemmner W. Wdr66 is a novel marker for risk stratification and involved in epithelial-mesenchymal transition of esophageal squamous cell carcinoma. BMC cancer. 2013;13:137. doi: 10.1186/1471-2407-13-137.

5. Dennis G, Jr., Sherman BT, Hosack DA, Yang J, Gao W, Lane HC, et al. DAVID: Database for Annotation, Visualization, and Integrated Discovery. Genome Biol. 2003;4(5):P3. PubMed PMID: 12734009.
6. Mi H, Dong Q, Muruganujan A, Gaudet P, Lewis S, Thomas PD. PANTHER version 7: improved phylogenetic trees, orthologs and collaboration with the Gene Ontology Consortium. Nucleic Acids Res. 2010;38(Database issue):D204-10. doi: 10.1093/nar/gkp1019. PubMed PMID: 20015972; PubMed Central PMCID: PMCPMC2808919.
7. Thomas PD, Kejariwal A, Guo N, Mi H, Campbell MJ, Muruganujan A, et al. Applications for protein sequence-function evolution data: mRNA/protein expression analysis and coding SNP scoring tools. Nucleic Acids Res. 2006;34(Web Server issue):W645-50. PubMed PMID: 16912992; PubMed Central PMCID: PMCPMC1538848.
